# Effect of heat shock on hot water plumbing microbiota and *Legionella pneumophila* control

**DOI:** 10.1186/s40168-018-0406-7

**Published:** 2018-02-09

**Authors:** Pan Ji, William J. Rhoads, Marc A. Edwards, Amy Pruden

**Affiliations:** Via Department of Civil and Environmental Engineering, Virginia Tech, Blacksburg, VA 24061 USA

**Keywords:** Heat shock, Hot water plumbing, Distal taps, Opportunistic pathogens—biofilm, 16S rRNA gene amplicon sequencing

## Abstract

**Background:**

Heat shock is a potential control strategy for *Legionella pneumophila* in hot water plumbing systems. However, it is not consistently effective, with little understanding of its influence on the broader plumbing microbiome. Here, we employed a lab-scale recirculating hot water plumbing rig to compare the pre- and post-“heat shock” (i.e., 40 → 60 → 40 °C) microbiota at distal taps. In addition, we used a second plumbing rig to represent a well-managed system at 60 °C and conducted a “control” sampling at 60 °C, subsequently reducing the temperature to 40 °C to observe the effects on *Legionella* and the microbiota under a simulated “thermal disruption” scenario.

**Results:**

According to 16S rRNA gene amplicon sequencing, in the heat shock scenario, there was no significant difference or statistically significant, but small, difference in the microbial community composition at the distal taps pre- versus post-heat shock (both biofilm and water; weighted and unweighted UniFrac distance matrices). While heat shock did lead to decreased total bacteria numbers at distal taps, it did not measurably alter the richness or evenness of the microbiota. Quantitative PCR measurements demonstrated that *L. pneumophila* relative abundance at distal taps also was not significantly different at 2-month post-heat shock relative to the pre-heat shock condition, while relative abundance of *Vermamoeba vermiformis*, a known *Legionella* host, did increase. In the thermal disruption scenario, relative abundance of planktonic *L. pneumophila* (quantitative PCR data) increased to levels comparable to those observed in the heat shock scenario within 2 months of switching long-term operation at 60 to 40 °C. Overall, water use frequency and water heater temperature set point exhibited a stronger effect than one-time heat shock on the microbial composition and *Legionella* levels at distal taps.

**Conclusions:**

While heat shock may be effective for instantaneous *Legionella* control and reduction in total bacteria numbers, water heater temperature set point and water use frequency are more promising factors for long-term *Legionella* and microbial community control, illustrating the importance of maintaining consistent elevated temperatures in the system relative to short-term heat shock.

**Electronic supplementary material:**

The online version of this article (10.1186/s40168-018-0406-7) contains supplementary material, which is available to authorized users.

## Background

Hot water systems are a key source of microbes to the human-occupied built environment and harbor distinct microbiota from that of influent potable water [1]. In particular, while influent cold water lines can be subject to significant seasonal variation [2], spatial and temporal patterns in the microbial community composition of hot water systems can be even more complex due to variable flow patterns and configurations [3], stagnation time of distal taps [4, 5], and temperature conditions [1]. Further, in-building hot water systems often involve storage equipment (e.g., water heater or hot water tank), which can serve as a reservoir for microbes and contribute to downstream warm water conditions conducive to microbial growth [6]. Elevated temperatures also accelerate disinfectant decay (e.g., chlorine, [7]) and predispose hot water systems to deteriorating microbial water quality. Such unique aspects of hot water systems, together with the inherent heterogeneity in domestic plumbing designs across different buildings, make it almost impossible to promote a unified control strategy for microbial regrowth.

Hot water systems are especially vulnerable to the growth of opportunistic pathogens (OPs), such as *Legionella pneumophila* and *Mycobacteria avium*. This emphasizes the critical role of design and operation for protecting public health, particularly when serving immunocompromised populations, such as in hospital settings. As early as 1987, hospital hot water tanks with temperature settings below 55 °C were identified as the primary source for nosocomial Legionnaire’s disease outbreaks [8, 9]. Notably, implementation of energy and water conservation features can unintentionally increase the risk of OP exposure [10], as was observed in a 400-bed university hospital in Sherbrooke, Canada, where elevated *L. pneumophila* growth in the hot water system was associated with the installation of a heat exchanger [11]. In residential homes, showers represent a routine source of potential exposure to aerosolized OPs. Importantly, inhalation of such aerosols is the primary route of infection, rather than ingestion, as is the emphasis of drinking water regulations [12].

Much attention has been directed to control measures for OPs in hot water systems, including thermal disinfection/heat shock [13], UV disinfection [14], on-site secondary disinfection (e.g., monochloramine [15]), and copper-silver ionization [16]. Still, there is a wide debate regarding the optimal choice for on-site OP control, with thermal disinfection or heat shock remaining one of the most widely accessible and feasible options for many building owners/residents. Thermal disinfection, or heat shock, typically involves setting the water heater temperature at a high level over a defined period of time and subsequently continuously or periodically flushing distal taps for a target duration at a minimal at-the-tap flushing temperature. Notably, there is a range of thermal disinfection or heat shock procedures defined by various professional and public health agencies with respect to several key elements (see Additional file 1: Table S1), including water heater temperature set point (60–77 °C) and flushing condition (continuous or periodic).

Control of OPs within hot water systems is inherently a matter of managing the microbial ecology [17], as OPs are native to the drinking water environment and thus not as readily eradicated as fecal pathogens. Further, the high surface area to volume ratio characteristic of domestic plumbing encourages biofilm development, where OPs, including *L. pneumophila*, benefit from a parasitic relationship with amoebae that enables their proliferation within the highly oligotrophic drinking water environment [18, 19]. However, the precise effects of heat shock for control of OPs (especially *L. pneumophila*) have not been systematically evaluated. Prior studies have examined *L. pneumophila* specifically [13, 20] and short-term response to heat shock (e.g., 7 days [21]) or heat-treated tap water [22]. Still, significant knowledge gaps remain with respect to long-term effects on (a) OPs within the context of the broader microbial community composition and (b) bulk water and biofilm phases and their interrelationship.

This study employed a heat shock protocol at the “mild” end of the spectrum, with water heater temperature set point elevated to 60 °C and periodic flushing at distal taps to maintain at-the-tap temperature > 55 °C for 30 min, to gain a sense of the physical effect of heat shock to the microbes within a temperature regime widely accessible to building owners and residents. Here, we employed a lab-scale recirculating hot water plumbing rig to compare the pre- and post-heat shock (i.e., 40 → 60 → 40 °C) microbiota at distal taps (“heat-shock” scenario). In addition, we used a second plumbing rig to represent a well-managed system maintaining elevated temperature throughout the recirculating line (60 °C), and reduced the temperature to 40 °C to observe the effects on *Legionella* and the microbiota (“thermal disruption” scenario). Effects of heat shock and thermal disruption were compared relative to those imparted by the water heater temperature set point, pipe orientation, and the water use frequency at the tap.

## Methods

### System setup and experimental design

Two identical hot water rigs were constructed to examine the impact of thermal conditions. Rig design has been previously described in detail [6, 23]. Each rig consisted of an electric water heater and recirculating pipe, with 18 distal taps comparing two pipe orientations (downward with little convective mixing vs slanted upward with enhanced convective mixing) and three water use frequencies in triplicate (high-, medium-, low-water use as 21, 3, 1 flushes/week, respectively). Prior to this study, both rigs had been acclimated with Blacksburg, VA, tap water for 15 months. Municipal chloramine residual was removed by passing water through three granular activated carbon filters in series [1, 24]. In addition, the upward-oriented pipes were tilted 30° from vertical 4-month pre-heat shock (2 months prior to the first sampling point) to induce convective mixing for comparison to downward oriented pipes without convective mixing.

### Heat shock and thermal disruption

Parallel comparison of the two rigs allowed examination of heat shock as a control measure relative to water heater temperature set point and flow conditions at the tap. Prior to imposing the shifts in thermal conditions associated with the present study, the “heat shock” (referred to as “control” in prior studies) and “thermal disruption” (referred to as “experimental” in prior studies) rigs had been maintained at water heater temperature set points of 40 and 60 °C, respectively, for 4 months [1, 6, 23]. Note that all temperatures cited herein can vary from the water heater temperature set point by ± 1–2 °C. To commence the present study, both rigs were set to 60 °C, and each set of distal taps were flushed intermittently to maintain water temperatures > 55 °C for approximately 30 min, targeting the guidance of the Association of Water Technologies [25] and Stout et al. [26] (summary of published heat shock treatment procedures summarized in Additional file 1: Table S1, adapted from Table 1 in [27]). While this did not constitute a substantive “heat shock” to the “thermal disruption” rig (given that it was already maintained at 60 °C), both rigs were subject to the same treatment to normalize the effects of flushing the distal lines at elevated temperature for an extended period of time. Post-heat shock, both water heater temperatures were set to 40 °C.

### Sample collection

**Fig. 1:**
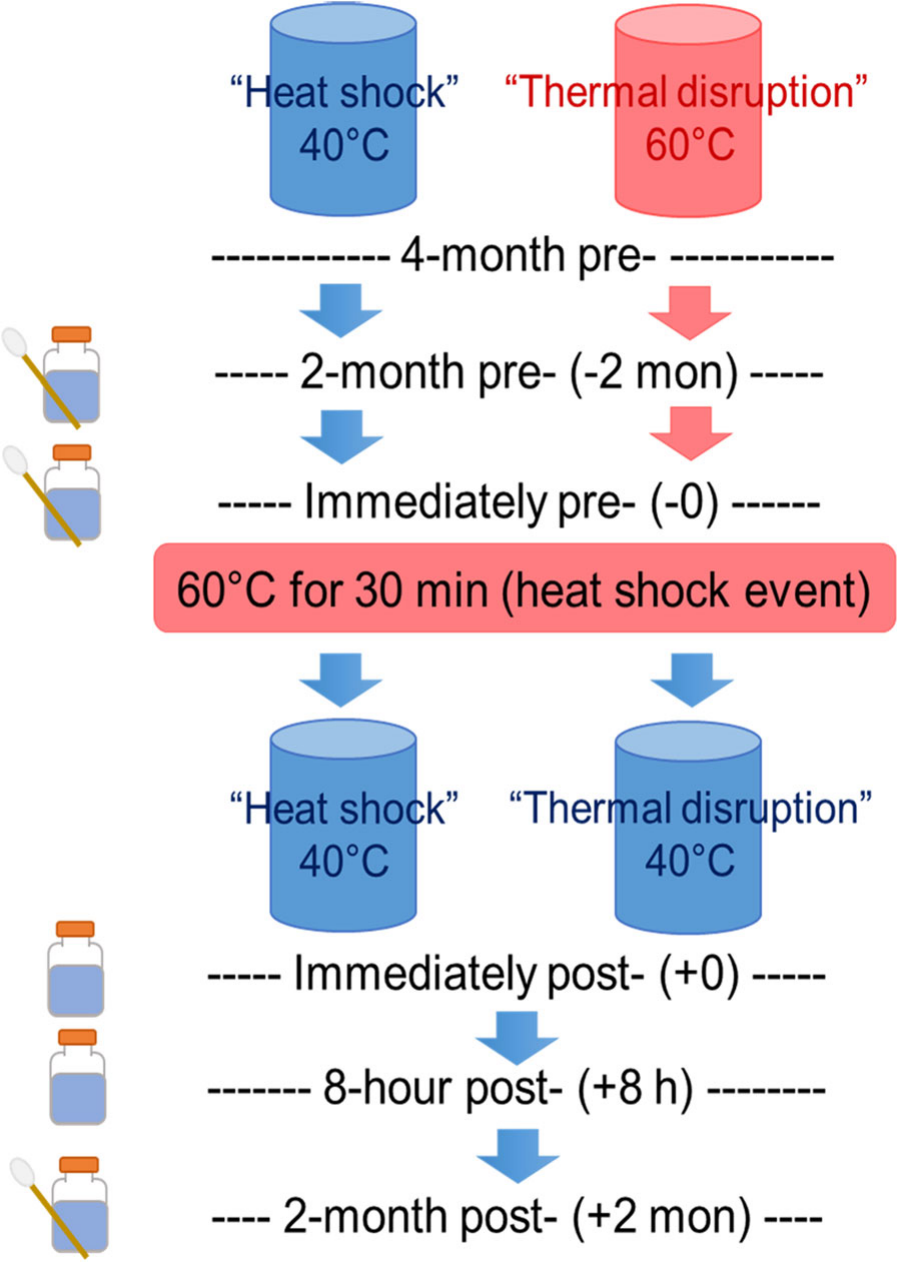
Experimental timeline. Time is relative to the heat shock event

First-flush bulk water (~ 500 mL) and biofilm (65-cm^2^ inner surface area swabbed) samples were collected at each sampling portal (influent, recirculating line and distal taps) 2-month pre- (i.e., time = − 2 mon), immediately pre- (time = -0), and 2-month post-heat shock (time = + 2 mon) (Fig. 1). Additional first-flush bulk water samples were collected immediately post- (+ 0) and 8-h post- (+ 8 h) heat shock (Fig. 1). The rationale for collecting these additional bulk water samples was to capture potential changes during the heat shock process (immediately post-heat shock) and after a modest stagnation time mimicking daily water use pattern (8-h post-heat shock). If not specified, the term “pre-heat shock samples” refers to samples taken at 2-month pre- and immediately pre-heat shock, while the term “post-heat shock samples” only includes samples taken at 2-month post-heat shock. Distal tap samples were typically collected at the end of the cyclical 8-h stagnation periods for each water use frequency.

### DNA extraction, qPCR, and 16S rRNA amplicon sequencing

#### DNA extraction

Bulk water samples were first concentrated onto sterile 0.22-μm pore size mixed-cellulose-ester filters (Millipore, Billerica, MA, USA). DNA extraction from the fragmented filters and cotton swabs followed the FastDNA Spin Kit (MP Biomedicals, Solon, OH, USA) manufacturer protocol.

#### Quantitative PCR

Quantitative polymerase chain reaction (qPCR) was applied to quantify the gene copy numbers of total bacteria (16S rRNA), *L. pneumophila*, *Mycobacterium* *avium* and *Vermamoeba vermiformis* (née *Hartmanella vermiformis*) using established protocols [28–32]. A dilution ratio of 1:10 was selected for all DNA extracts to balance inhibition and detection. Each sample was analyzed in triplicate, where at least two positive reads were scored as a positive detection of a given gene. To determine relative abundances, *L. pneumophila*, *M. avium*, and *V. vermiformis* gene copy numbers were normalized to total bacterial 16S rRNA gene copy numbers.

#### Amplicon sequencing

Sample preparation for 16S rRNA gene amplicon sequencing followed the online Earth Microbiome Project protocol [33] using the 515F/926R primer pair targeting the V4 and V5 regions of the 16S rRNA gene. Minor differences include using molecular grade water (Quality Biological, Gaithersburg, MD, USA) and pooling PCR products on an equal mass basis of 200 ng. Illumina amplicon sequencing was performed on MiSeq platform at the Biocomplexity Institute at Virginia Tech (paired-end 300 bp reads using MiSeq Kit V3).

### Amplicon sequencing data analysis

Demultiplexed amplicon sequencing data were retrieved and processed using the PANDAseq assembler [34] to stitch the paired-end reads with the criteria that the stitched read length should be between 372 and 375 bp and the threshold score of at least 0.80. Chimera-free sequences (USEARCH v6.1 [35], reference-based chimera detection using Greengene database v13_8 [36]) were subject to de novo operational taxonomy unit (OTU) picking strategy (pick_de_novo_otus.py) at 0.97 similarity (UCLUST [35]) in QIIME 1.8.0 [37] referencing Greengene database v13_8 [36]. Sequences were aligned using PyNAST [38]. Taxonomy was assigned using RDP Classifier 2.2 [39]. An approximately maximum-likelihood phylogenetic tree was constructed using FastTree 2.1.3 [40]. Further, singletons (OTU with one sequence across the entire OTU table) and organelle OTUs (chloroplast and mitochondria) were removed from the downstream analysis. A total of 10, 313, 752 sequences were retained for all 323 samples with a median value of 31, 946 sequences per sample (min 5, 210; max 111, 018). The cleaned OTU table was then rarefied 100 times to a sequencing depth of 5200, from which alpha diversity (Chao 1 index for richness; Gini index for evenness) and beta diversity (weighted and unweighted UniFrac distance matrices, [41]) was measured. Difference in alpha diversity (Chao 1 index) across different time points pre- and post-heat shock was examined via Kruskal-Wallis test, with Nemenyi test for pairwise comparisons (package “PMCMR” version 4.1 [42]). Permutational multivariate analysis of variance (Adonis [43]) was applied to the average weighted and unweighted UniFrac distance matrices as a measurement of difference in group means. Complementary multivariate homogeneity of group dispersions analysis (betadisper [44]) was applied to evaluate the within-group variations (package “vegan” version 2.3-0 [45]), where homogeneity of dispersion among groups is an assumption for Adonis. All sequence data have been deposited in QIITA under study ID 10504 and European Nucleotide Archive (ENA) under accession number PRJEB22241.

#### qPCR data analysis

Gene copy numbers determined by qPCR were converted to concentration in water/biofilm based on the volume/area sampled. Gene copy numbers were normalized with total bacterial 16S rRNA gene copy numbers as a proxy indicator of relative abundance. All statistical analyses, including linear regression and Spearman correlation analysis, were conducted in R (version 3.3.1 [46]).

## Results

### Heat shock imposed limited impact on microbial composition of biofilm

For the biofilm phase, weighted and unweighted UniFrac distance matrices yielded highly similar trends (Table 1a, b; weighted UniFrac see Additional file 2: Figure S1). In the “heat shock” rig, comparison of pre- versus post-heat shock biofilm samples yielded either statistically significant, but minute, difference (2-month pre- vs 2-month post-, weighted UniFrac, *R*^2^_Adonis_ = 0.0760, *P*_Adonis_ = 0.020) or no statistically significant difference (immediately pre- vs 2-month post-, weighted UniFrac, *R*^2^_Adonis_ = 0.0623, *P*_Adonis_ = 0.056; Table 1a) in microbial community composition. In the “thermal disruption” rig, post-heat shock (40 °C) biofilm samples appeared to be distinct from those pre-heat shock (60 °C) (weighted UniFrac, *R*^2^_Adonis_ = 0.2104/0.2193, *P*_Adonis_ = 0.001, Table 1b), where temporal variation within pre-heat shock biofilm samples were insignificant (weighted UniFrac, *R*^2^_Adonis_ = 0.0202, *P*_Adonis_ = 0.566; Table 1b). Together, these results are suggestive that the water heater set point had a stronger influence than the prior heat shock history (Table 1a, b).

**Table 1:** Statistical comparison of samples collected pre- and post-heat shock (permutation = 999)

	Weighted UniFrac			Unweighted UniFrac		
	*R* ^2^_Adonis_	*P*_Adonis_	*P*_Betadisp_	*R* ^2^_Adonis_	*P*_Adonis_	*P*_Betadisp_
a. “Heat shock” rig, biofilm samples						
2-month pre- (19) vs 2-month post- (19)	**0.0760**	**0.020**	0.919	**0.0737**	**0.001**	0.430
2-month pre- (19) vs immediately pre- (18)	0.0310	0.307	0.257	0.0352	0.098	0.901
Immediately pre- (18) vs 2-month post- (19)	0.0623	0.056	0.297	**0.0553**	**0.001**	0.416
b. “Thermal disruption” rig, biofilm samples						
2-month pre- (19) vs 2-month post- (19)	**0.2104**	**0.001**	0.169	**0.1525**	**0.001**	**0.001**
2-month pre- (19) vs immediately pre- (18)	0.0202	0.566	0.781	0.0292	0.299	0.995
Immediately pre- (18) vs 2-month post- (19)	**0.2193**	**0.001**	0.128	**0.1504**	**0.001**	**0.001**
c. “Heat shock” rig, water samples						
2-month pre- (20) vs 2-month post- (21)	**0.1434**	**0.003**	**0.018**	**0.0789**	**0.001**	**0.019**
2-month pre- (20) vs immediately pre- (18)	**0.2274**	**0.001**	0.400	**0.0598**	**0.001**	0.604
Immediately pre- (18) vs 2-month post- (21)	**0.2209**	**0.001**	**0.006**	**0.0763**	**0.001**	**0.005**
d. “Thermal disruption” rig, water samples						
2-month pre- (20) vs 2-month post- (21)	**0.3808**	**0.001**	0.340	**0.1737**	**0.001**	0.499
2-month pre- (20) vs immediately pre- (18)	**0.1038**	**0.009**	0.471	**0.0455**	**0.003**	0.478
Immediately pre- (18) vs 2-month post- (21)	**0.2498**	**0.001**	0.056	**0.1593**	**0.001**	0.979

Bold font indicates statistically significant value (*P* < 0.05)

### Heat shock imposed limited impact on microbial composition of bulk water

For the bulk water phase, the impacts of the experimental conditions were generally more apparent when applying the weighted (Additional file 2: Figure S1), than the unweighted, UniFrac distance matrix (Table 1c, d), indicating a stronger effect in terms of relative abundance than the occurrence of OTUs. In the “heat shock” rig, although the 2-month post-heat shock microbial community structure was significantly different from that of the pre-heat shock condition (weighted UniFrac, *R*^2^_Adonis_ = 0.1434/0.2209, *P*_Adonis_ = 0.003/0.001), the difference observed was no greater than that associated with temporal variation of the two pre-heat shock samplings (2-month pre- vs immediately pre-, weighted UniFrac, *R*^2^_Adonis_ = 0.2274, *P*_Adonis_ = 0.001, Table 1c). In the “thermal disruption” rig, there was a much sharper difference between the pre- and post-temperature drop water samples (weighted UniFrac, *R*^2^_Adonis_ = 0.3808/0.2498, *P*_Adonis_ = 0.001) than between the two water samples collected at the 60 °C set point before the temperature drop to 40 °C (weighted UniFrac, *R*^2^_Adonis_ = 0.1038, *P*_Adonis_ = 0.009). This again points to the water heater set point as the dominant factor shaping the microbial community structure.

Eight-hour stagnation (immediately post- vs 8-h post-) was found to incur a statistically significant, but small, change in microbial composition based on both weighted (*R*^2^_Adonis_ = 0.0774, *P*_Adonis_ = 0.001) and unweighted (*R*^2^_Adonis_ = 0.0371, *P*_Adonis_ = 0.001) UniFrac distances (Additional file 3: Table S2).

### Effects of heat shock on microbial diversity

In the “heat shock” rig, when comparing the distal tap microbiome pre- versus post-heat shock, alpha diversity indicated no obvious change either in terms of richness (Chao 1 index, Additional file 4: Figure S2) in bulk water phase (immediately pre- vs immediately post-, *P* = 0.976; immediately pre- vs 8-h post-, *P* = 0.991) or evenness (Gini index, Additional file 5: Figure S3) in either biofilm (2-month pre- vs 2-month post-, *P* < 0.001, Gini index range 0.993–0.999 indicating limited difference) or bulk water (immediately pre- vs immediately post-, *P* = 0.928; immediately pre- vs 8-h post-, *P* = 1.000) phases. The decrease of richness (Chao 1 index) in the biofilm phase after the heat shock treatment (Additional file 4: Figure S2) was no greater than that observed in influent biofilm (1695, 1438, 1015 for 2-month pre-, immediately pre-, and 2-month post-, respectively, in influent biofilm), which suggests that temporal factors were the more plausible driver than the heat shock treatment. The limited effect of heat shock on microbial diversity was further corroborated by the near-perfect correlation of the relative abundances of individual OTUs pre- and post-heat shock (Additional file 6: Figure S4).

### Comparison of heat shock versus thermal disruption

**Fig. 2:**
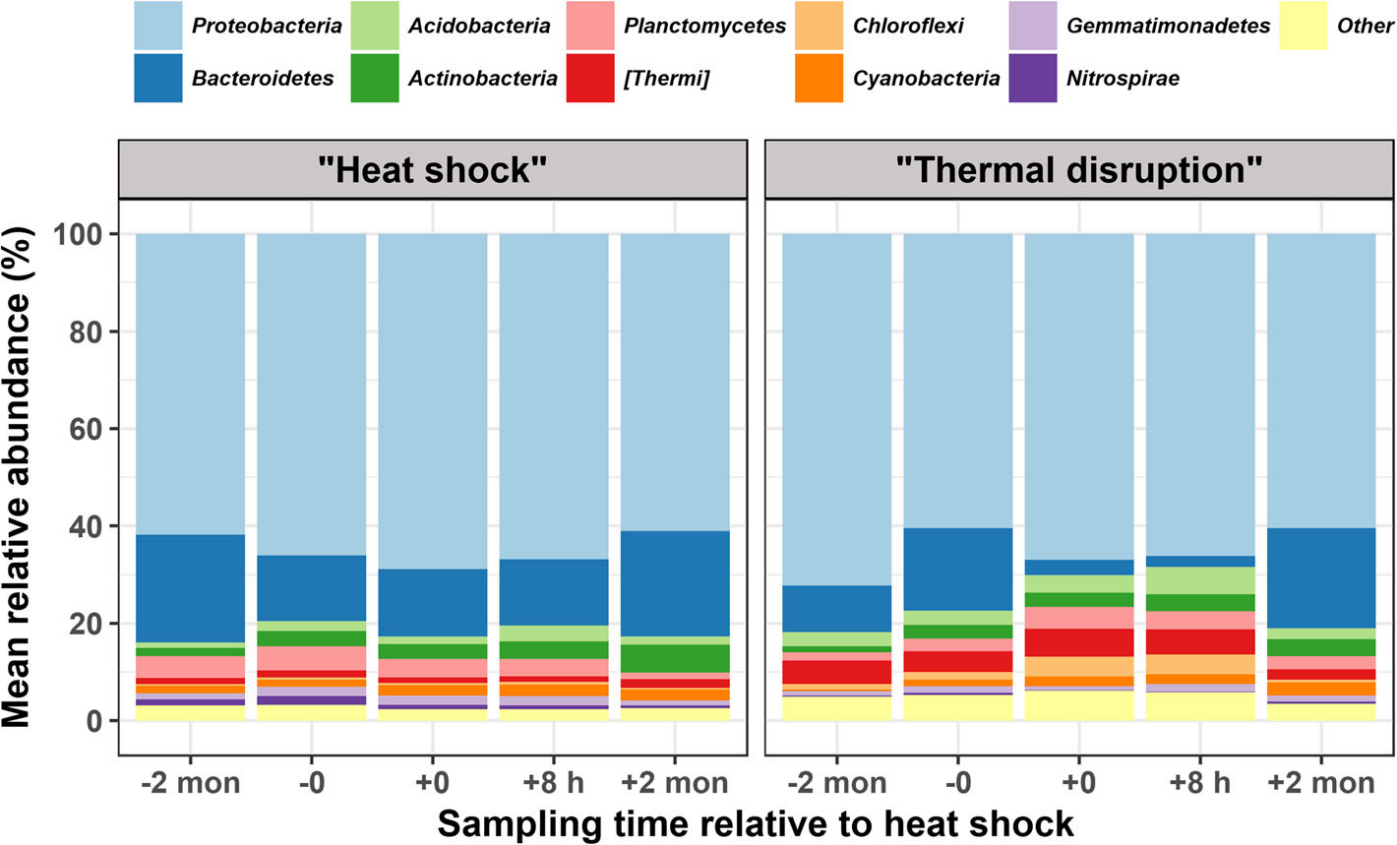
Top 10 abundant phyla in distal tap water samples across sampling time. Abundant phyla were determined based on all samples combined and ranked in descending order by relative abundance from the top to the bottom of each bar in the figure. Remaining phyla were aggregated as “other” and presented at the bottom of each bar

A key question is whether adjusting the water heater temperature directly shapes the microbial community structure and essentially overrides the influence of previous temperature regimes. To evaluate this, the “thermal disruption” and “heat shock” rigs were compared after 2 months of maintaining both rigs at 40 °C following the heat shock. While the distal tap microbial communities were still distinct between the two rigs (both phases), the differences were smaller than those observed when comparing the pre-heat shock conditions, trending towards convergence (Table 2). Indeed, the distal tap bulk water microbiota from the two rigs remained distinct immediately (*R*^2^_Adonis_ = 0.7772, *P*_Adonis_ = 0.001) and 8-h post-heat shock (*R*^2^_Adonis_ = 0.6692, *P*_Adonis_ = 0.001), where the *Bacteroidetes* phylum showed much lower relative abundance in the “thermal disruption” rig than in the “heat shock” rig (Fig. 2).

**Table 2:** Statistical comparison on the relative impact of rig, pipe orientation, and water use frequency. Weighted UniFrac distance matrix was applied for Adonis and Betadisp test with permutation = 999 ({vegan}, *R*)

	Rig			Pipe orientation			Water use frequency		
	*R* ^2^_Adonis_	*P*_Adonis_	*P*_Betadisp_	*R* ^2^_Adonis_	*P*_Adonis_	*P*_Betadisp_	*R* ^2^_Adonis_	*P*_Adonis_	*P*_Betadisp_
a. Biofilm samples									
2-month pre- (36)	**0.2917**	**0.001**	0.261	**0.1160**	**0.004**	0.487	**0.1508**	**0.006**	0.290
Immediately pre- (35)	**0.2082**	**0.001**	**0.014**	**0.1782**	**0.001**	**0.001**	**0.1897**	**0.003**	0.873
2-month post- (36)	**0.1614**	**0.001**	**0.001**	**0.1925**	**0.001**	**0.024**	**0.1121**	**0.031**	0.383
b. Bulk water samples									
2-month pre- (36)	**0.4311**	**0.001**	**0.012**	**0.1580**	**0.010**	0.308	0.1018	0.104	0.466
Immediately pre- (34)	**0.2937**	**0.001**	**0.001**	**0.0944**	**0.016**	0.225	0.1125	0.056	0.282
Immediately post- (36)	**0.7772**	**0.001**	0.833	0.0116	0.645	0.714	0.0388	0.550	0.935
8-h post- (36)	**0.6692**	**0.001**	0.833	0.0253	0.364	0.965	0.0337	0.655	0.770
2-month post- (36)	**0.1076**	**0.013**	0.896	0.0343	0.298	0.909	**0.5350**	**0.001**	0.051

Bold font indicates statistically significant value (*P* < 0.05)

### Influence of water heater temperature set point, pipe orientation, and water use frequency

The relative importance of rig (representing water heater temperature set point difference), pipe orientation, and water use frequency on microbial community composition, as represented by dissimilarity matrices, varied with sampling time and phase (Table 2). For instance, at 2-month post-heat shock (both rigs at 40 °C), pipe orientation was the dominant factor for the biofilm phase, but had no significant effect for the bulk water phase (Table 2). Instead, water use frequency was the dominant factor shaping the bulk water microbial community composition. There also appeared to be potential synergistic factors between pipe orientation and water use frequency. For example, downward pipe orientation magnified the influence of water use frequency on the microbial composition of the biofilm (Additional file 7: Figure S5; weighted UniFrac, downward *n* = 54, *R*^2^_Adonis_ = 0.2878, *P*_Adonis_ = 0.001 vs upward *n* = 53, *R*^2^_Adonis_ = 0.0870, *P*_Adonis_ = 0.019), likely by altering relative abundance more than occurrence of individual OTUs (unweighted UniFrac, downward *R*^2^_Adonis_ = 0.0743, *P*_Adonis_ = 0.003 vs upward *R*^2^_Adonis_ = 0.0661, *P*_Adonis_ = 0.002). No such obvious interactions between pipe orientation and water use frequency were observed in bulk water phase.

Notably, among the “heat shock” rig distal tap biofilm samples (*n* = 53), pipe orientation (*R*^2^_Adonis_ = 0.2378, *P*_Adonis_ = 0.001) and water use frequency (*R*^2^_Adonis_ = 0.2026, *P*_Adonis_ = 0.001) appeared to be more influential than heat shock (pre- vs post-heat shock, *R*^2^_Adonis_ = 0.0662, *P*_Adonis_ = 0.01), although all three factors indicated small effects when considering the unweighted UniFrac distance matrix (*R*^2^_Adonis_ = 0.0574/0.0826/0.0877, *P*_Adonis_ = 0.001).

### Impact of heat shock on *Legionella* spp. and *Mycobacterium* spp. based on 16S rRNA amplicon sequencing

**Fig. 3:**
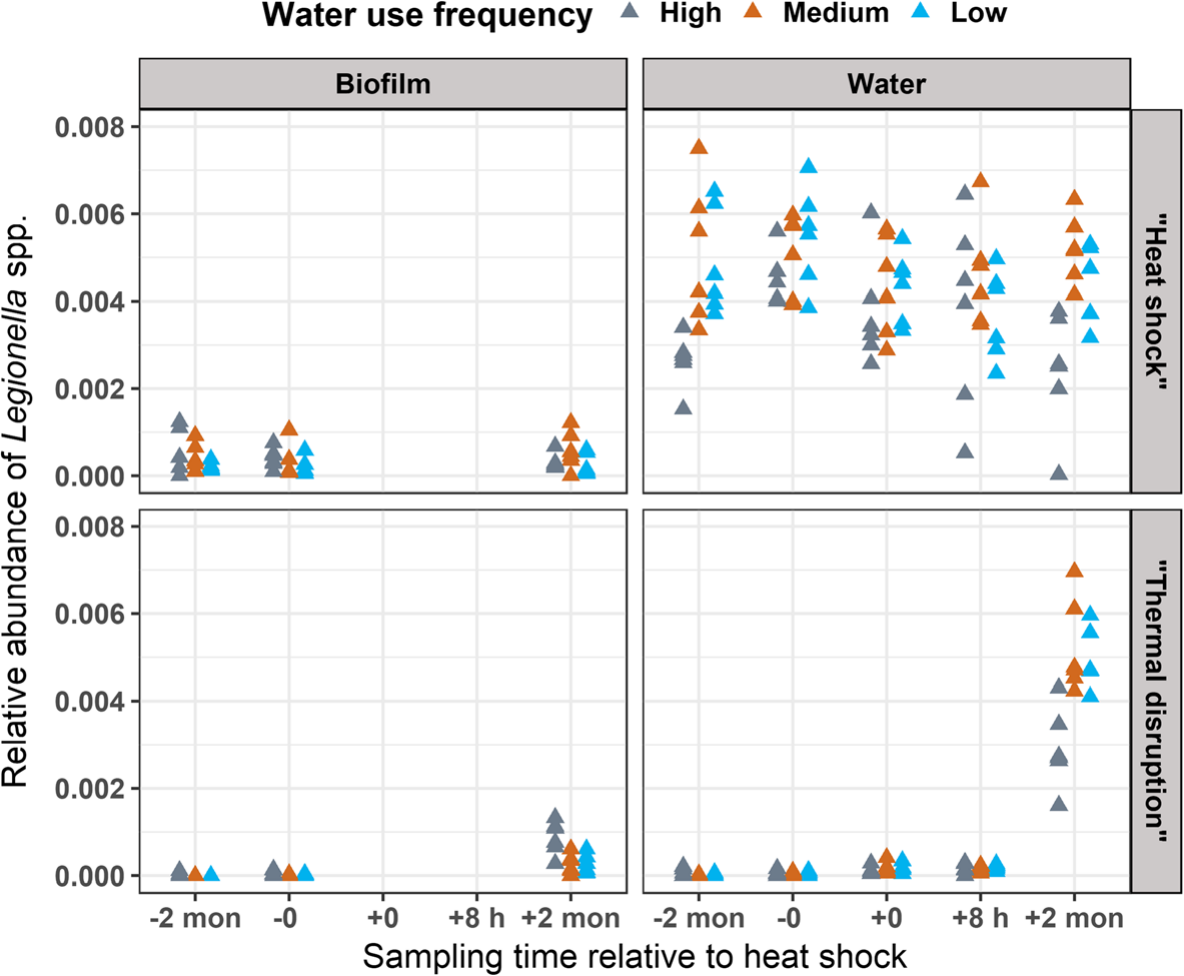
Relative abundance of *Legionella* spp. in distal tap samples. Relative abundance was calculated from 16S rRNA gene amplicon sequencing data

**Fig. 4:**
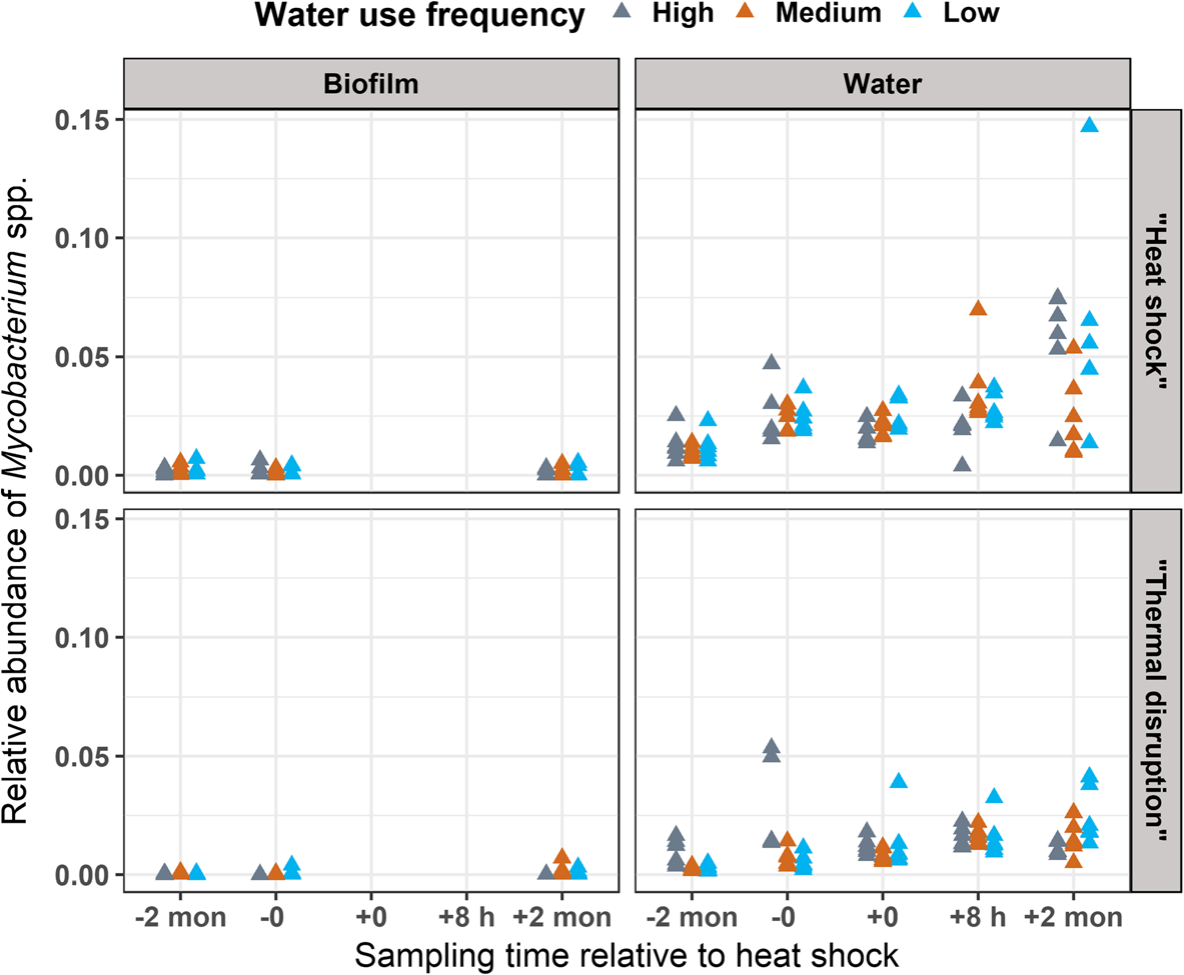
Relative abundance of *Mycobacterium* spp. in distal tap samples. Relative abundance data was calculated from 16S rRNA gene amplicon sequencing data

The relative abundance of *Legionella* spp. at the distal tap remained relatively constant in both the biofilm and bulk water across all time points in the “heat shock” rig. By contrast, *Legionella* spp. relative abundance increased markedly in both phases of the “thermal disruption” rig following operation for 2 months at the reduced temperature of 40 °C (both phases, Fig. 3). High water use frequency (shortest stagnation period) was associated with a low relative abundance of *Legionella* spp. in pre- and post-heat shock bulk water samples from the “heat shock” rig and post-heat shock bulk water samples from the “thermal disruption” rig. Interestingly, *Mycobacterium* spp. were enriched post-heat shock in the bulk water phase of the “heat shock” rig (both for high and low water use frequency, Fig. 4; no obvious trend in influent). *Mycobacterium* spp. did not appear to be as sensitive to water heater temperature set point as *Legionella* spp. (e.g., 60 °C pre-heat shock vs 40 °C post-heat shock samples in the “thermal disruption” rig). The “thermal disruption” rig was also associated with lower planktonic *Mycobacterium* relative abundance relative to the “heat shock” rig, suggesting that longer-term operation at 60 °C may have kept levels lower, even after dropping to 40 °C for 2 months.

### Impact of heat shock on total bacteria, *L. pneumophila*, *V. vermiformis*, and *M. avium* gene copy numbers by qPCR

**Fig. 5:**
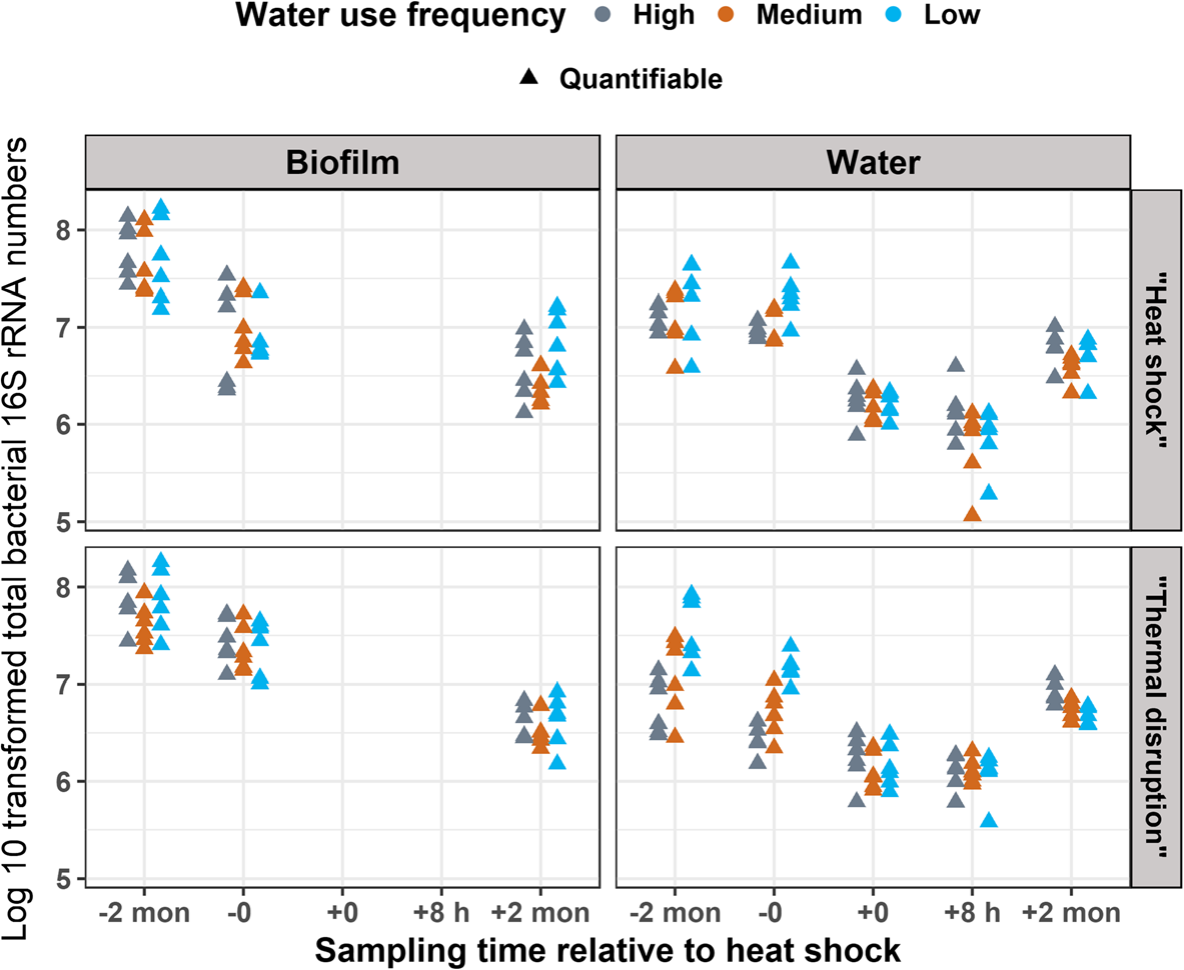
Total bacterial gene copy numbers in distal tap samples by qPCR. The gene copy numbers were log 10 transformed

**Fig. 6:**
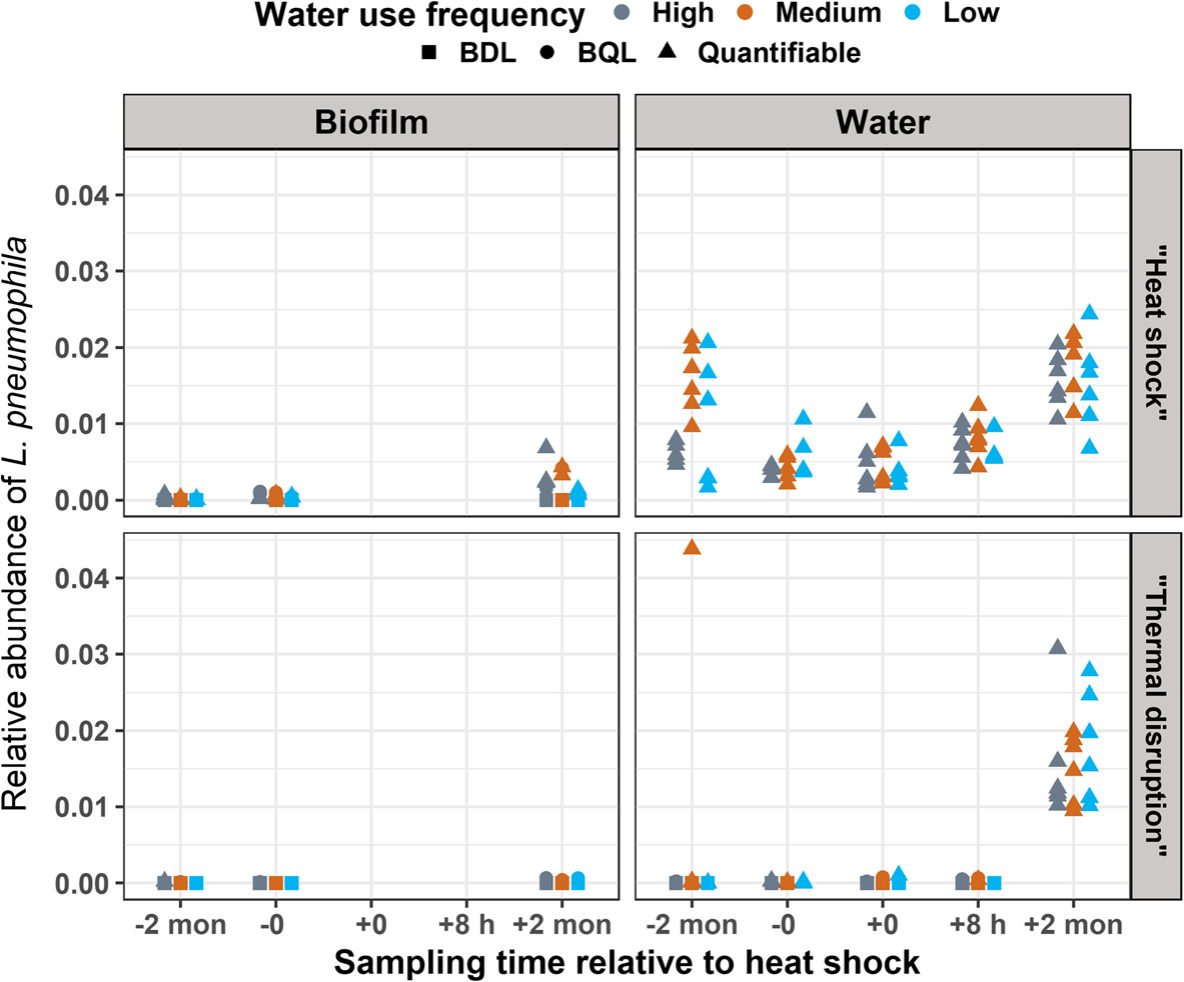
Relative abundance of *Legionella pneumophila* in distal tap samples by qPCR

Quantitative PCR measurements indicated lowest total bacteria numbers at 2-month post-heat shock in both rigs in both biofilm and bulk water (Fig. 5). In the “heat shock” rig, increased relative abundance (normalized to the 16S rRNA gene copy numbers) of *L. pneumophila* was observed in the biofilm following heat shock (Fig. 6). It should be noted, however, that *L. pneumophila* relative abundance was variable in the bulk water during the two baseline samplings prior to the heat shock (2-month pre- and immediately pre-heat shock). Relative abundance of *L. pneumophila* increased in the bulk water 2-month post-heat shock relative to immediately pre-heat shock but was comparable to levels 2-month pre-heat shock.

In the “thermal disruption” rig, decreased water heater temperature had no apparent effect on the relative abundance of *L. pneumophila* in the biofilm of distal taps, with consistently low levels observed pre- and post-heat shock (Fig. 6). However, in the bulk water, the relative abundance of *L. pneumophila* increased substantially 2 months after lowering the temperature to 40 °C, to a level comparable to those observed after 2 months at 40 °C post-heat shock in the “heat shock” rig (Fig. 6).

**Fig. 7:**
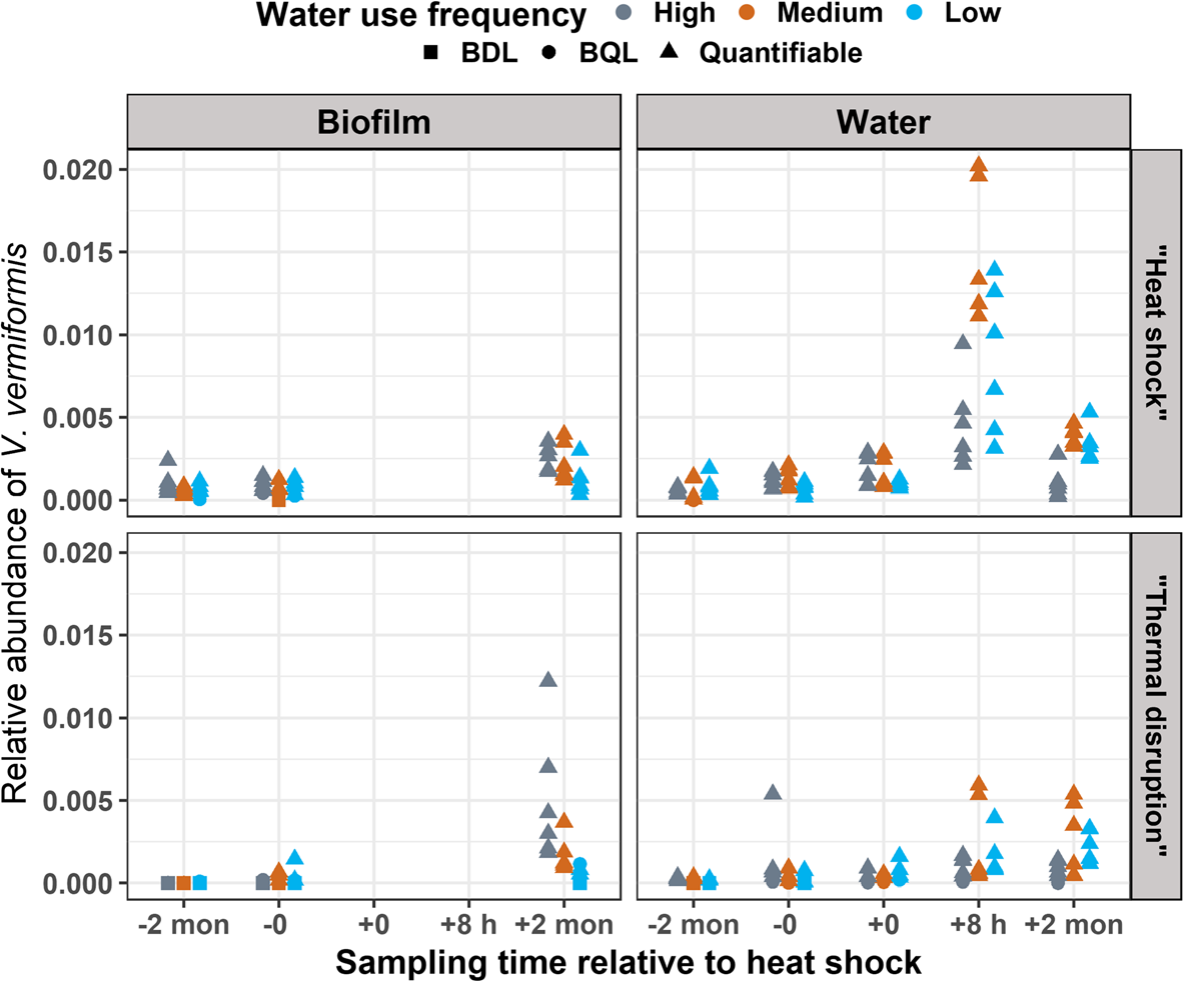
Relative abundance of *Vermamoeba vermiformis* in distal tap samples by qPCR

Post-heat shock samples tended to harbor higher relative abundance of *V. vermiformis* compared to pre-heat shock samples (both phases, both rigs, Fig. 7), although the opposite trend was observed in absolute numbers of *V. vermiformis* genes in the biofilm of the “heat shock” rig (Additional file 8: Figure S6). In the “heat shock” rig, distal tap bulk water achieved peak relative abundance of *V. vermiformis* at 8-h post-heat shock, which is likely due to lowest total bacterial gene copy numbers (Fig. 5) rather than corresponding peak *V. vermiformis* gene copy numbers (Additional file 8: Figure S6). Interestingly, in post-heat shock samples, the water use frequency associated with lowest relative abundance of *V. vermiformis* seemed to bifurcate between biofilm (low water use frequency) and bulk water (high water use frequency) conditions.

**Fig. 8:**
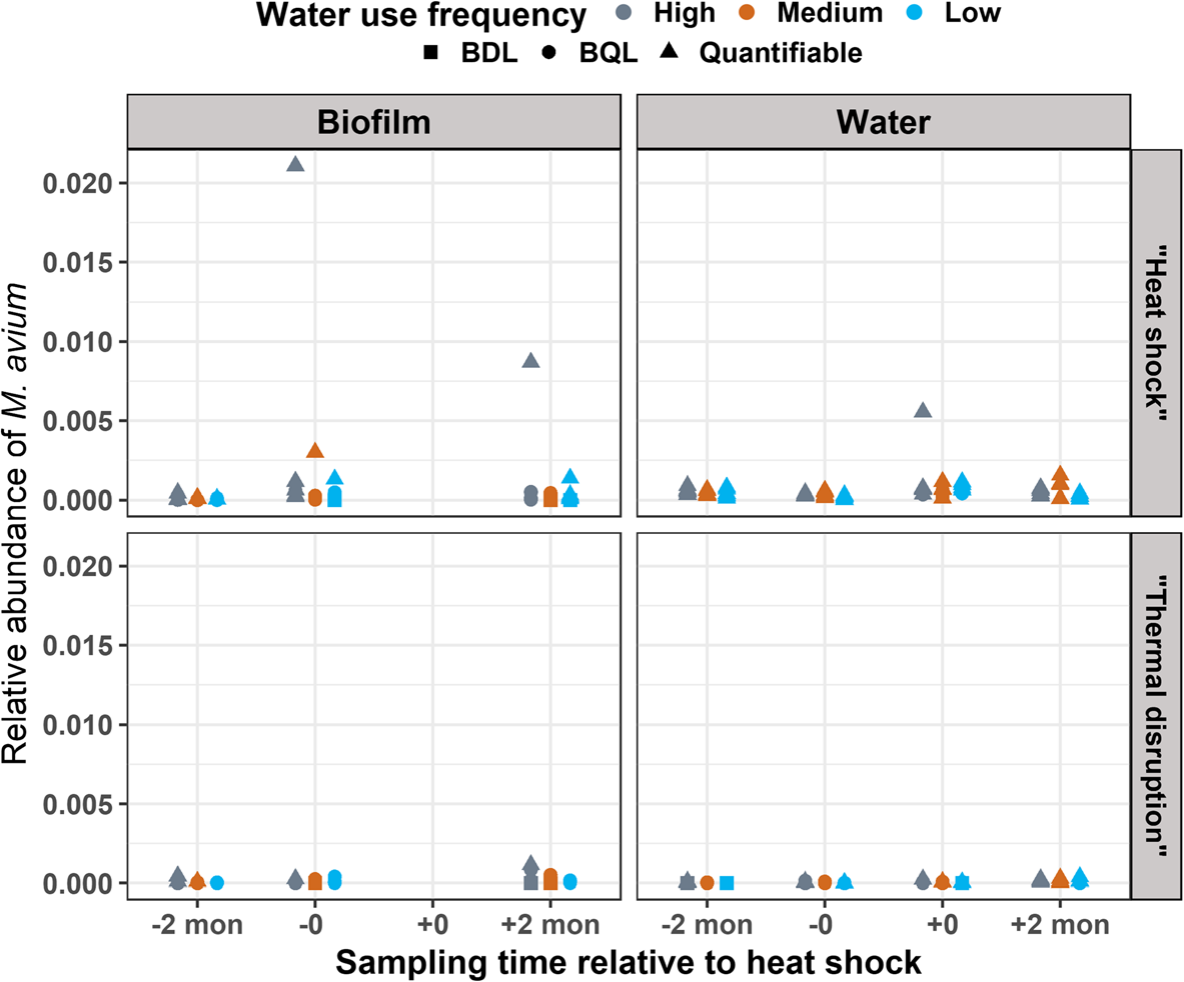
Relative abundance of *Mycobacterium avium* in distal tap samples by qPCR. Samples at 8-h post-heat shock were not included

Relative abundance of *M. avium* was consistently low (< 0.5%) in both phases of both rigs, except for three high “outlier” values and indicated no obvious trend with respect to heat shock (Fig. 8). The high “outlier” values appeared to be a mixed result of decreased total bacteria numbers (ranking 241, 249, and 180 out of a total of 249 samples in descending order) and/or elevated *M. avium* numbers (ranking 20, 66, and 4 out of a total of 249 samples in descending order; Additional file 9: Figure S7). The top two highest “outlier” values were from biofilm samples collected at the same distal tap (downward oriented, high water use frequency pipe in the “heat shock” rig, at immediately pre- and 2-month post-heat shock). The third highest “outlier” value was from a water sample collected at a distal tap (upward oriented, high water use frequency pipe in the “heat shock” rig, at immediately post-heat shock) with high *M. avium* gene copy numbers noted pre-heat shock. Notably, all three “outlier” values were associated with the “heat shock” rig and high water use frequencies, likely suggesting potential conditions which would enrich *M. avium*.

**Fig. 9:**
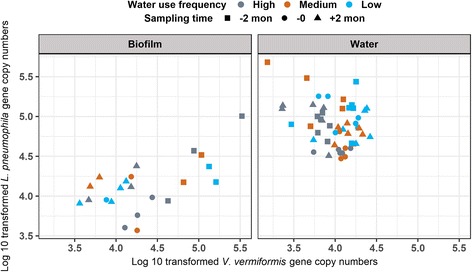
Relationship between *Legionella pneumophila* gene copy number and *Vermamoeba vermiformis* gene copy number. Samples included for analysis were (1) distal tap samples with (2) water heater temperature set point at 40 °C and (3) collected at 2-month pre-, immediately pre-, and 2-month post-heat shock, who had (4) quantifiable *L. pneumophila* and *V. vermiformis* numbers by qPCR. Both gene copy numbers were log-transformed with base 10

Combining data from the 40 °C conditions, 91 distal tap samples had quantifiable *L. pneumophila* and *V. vermiformis* numbers. Spearman correlation analysis indicated a positive association between *L. pneumophila* and *V. vermiformis* numbers in the biofilm (Spearman’s rank correlation coefficient = 0.48), while a negative association was noted in the bulk water (Spearman’s rank correlation coefficient = − 0.19, Fig. 9), though it was not visually apparent.

## Discussion

Heat shock alone, as employed in this study at the mild end of the spectrum of published methodologies, did not substantially influence the distal tap microbiota in terms of richness (Chao 1 index), evenness (Gini index), OTU counts, or microbial community dissimilarity patterns. The consistent trends based both on weighted and unweighted UniFrac distance matrices further demonstrated no apparent bias of heat shock affect towards abundant versus rare OTUs. At 2-month post-heat shock, the relative abundance of OPs and associated amoeba hosts either remained constant or increased slightly compared to pre-heat shock levels. Relative to the heat shock procedure as applied in the current study, water heater temperature set point and water use frequency each appeared to be more influential in terms of microbial community composition and *Legionella* control.

While from a microbial perspective, a sudden increase by 20 °C is certainly a “shock,” the null effect observed on biofilm microbiota could possibly be attributed to microbial resistance or resilience. The failure of the heat shock to disturb established biofilm microbiota is consistent with what has been called the “resistance” or insusceptibility scenario [47], suggesting ineffectiveness of the applied heat shock procedure as a microbiota control measure. Alternatively, in what might be called a “resilience” scenario, the microbial composition in the biofilm phase was indeed shifted by the heat shock, yet recovered close to pre-heat shock status within the subsequent 2 months. In this scenario, heat shock could effectively control biofilm microbiota if applied at an appropriate frequency. A previous study using a pilot-scale hot water distribution system [21] found that heat shock (water heater temperature set point at 70 °C and flushing of all taps for 30 min) conferred the most transitory effect on microbiota in the biofilm phase. In one of the two heat shock events where change in microbial composition was observed post-heat shock, the change was only observed on days 1, 3, and 7, sharing highly similar biofilm microbial composition to that of the non-heat-treatment conditions. Interestingly, in the present study where repeated swabbing of the same surface area for biofilm collection was employed with time, the composition of the biofilm was still highly stable in spite of different temperature regimes. This further underscores the conclusion that biofilms were relatively stable in the current study.

Unlike the relatively “stationary” biofilm phase where the influence of prior temperature regimes may be more long-lasting, the impact of the heat shock on the bulk water phase is expected to be more “transient.” When comparing bulk water immediately pre- and immediately post-heat shock in the “heat shock” rig, direct impacts were noted in terms of shifts in relative abundance, rather than occurrences of individual OTUs (Additional file 3: Table S2; Fig. 2). At 2-month post-heat shock, changes in bulk water microbial composition were minor and comparable to natural background fluctuation prior to the heat shock. With a simplified model of assuming complete mixing in the water heater and recirculating line, while ignoring microbial activity (i.e., growth and death), the bulk water affected by heat shock should be washed out of the system and thus not represent direct effects at 2-month post-heat shock (the proportion of water directly impacted by heat shock remained < 1 × 10^−15^, Additional file 10: Table S3). Indeed, microbes favored by heat shock theoretically would be subject to “wash out” (i.e., water use) following the loss of their thermophilic niche. Still, long-term influence of heat shock on the bulk water phase is possible via seeding of the bulk water from biofilm during stagnation, to the extent the biofilm phase was impacted in the longer term. However, this scenario is not as likely in this study given that there was limited influence of heat shock on the biofilm microbiota.

In this study, water heater temperature set point (60 vs 40 °C) and water use frequency appeared to be more influential than heat shock alone, both in terms of effect on the microbial community composition and control of *Legionella*. Ideally, a heat shock procedure achieves a sufficiently high water heater temperature set point (e.g., as high as > 70 °C) to exert a lethal effect on microbes throughout the system under continuous flow conditions, whereas the present study focuses on the mild end of the heat shock spectrum and may represent a “worst case scenario” of lack of observable benefit. Bédard et al. [13] applied a thermal disinfection procedure by maintaining the temperature setting above 70 °C for 1 h and flushing at the point-of-use for > 7 min in a 400-bed university hospital in Canada. They found that in one of the two hot water systems examined, thermal disinfection failed to affect the planktonic culturable *L. pneumophila* levels, where enhanced long-term thermal regime was a more important control measure than thermal disinfection. From a microbial ecology point of view, we speculate that the ineffectiveness of heat shock on *L. pneumophila* control is associated with its inability to affect distal tap microbial communities, even 2-month post-heat shock. Despite the absence of a uniform trend, all three high “outlier” values of *M. avium* relative abundance (by qPCR) were associated with high water use frequency and the “heat shock” rig, which was largely maintained at an inferior water heater temperature set point of 40 °C. It has been hypothesized in a previous study that higher water use frequency delivers more nutrients to distal taps within the same period, which can encourage OP growth under conditions of low water heater temperature set points and no disinfectant residual [24].

It should be emphasized that heat shock might unintentionally exacerbate OP exposure. First, we suspect that heat shock could induce a competitive advantage for OPs in the long term, even if they are reduced in the short term, as in the case of heat-pretreatment to select for more *Legionella* versus other bacteria prior to culturing [48]. Consistent with this hypothesis, heat shock did effectively decrease the total bacteria numbers, which could have contributed to lower OPs levels. However, *L. pneumophila* tended to recover quicker (Additional file 11: Figure S8) than total bacteria (Fig. 5), and become more enriched with time, especially in the bulk water phase (“heat shock” rig, Fig. 6). This is consistent with the findings of a stagnant-water model study [22], in which heat-treated tap water (60 °C for 30 min, subsequently cooled down to room temperature) contributed to elevated *L. pneumophila* levels in both biofilm and water phases compared to untreated tap water. It is of future research interest to determine how long it takes the total bacteria to be restored to pre-heat shock levels and whether the relative abundance of *L. pneumophila* will remain elevated during this process (i.e., increased *L. pneumophila* exposure risk). *V. vermoformis* was enriched post-heat shock mainly due to decreased total bacteria numbers (Fig. 5) rather than decreased *V. vermiformis* gene copy numbers (Additional file 8: Figure S6), potentially as an indicator of their feeding on the bacteria. *V. vermiformis* and other amoebae are known for their crucial role in *L. pneumophila*’s life cycle [49], higher relative abundance of which would have likely contributed to the increased levels of *L. pneumophila*. Second, heat shock might not be a sustainable method for engineering control of the microbiota: as each application would likely enhance the resistance of biofilm microbiota to the standard heat shock procedure, similar to the case of drought on the tropical forest soil microbial communities [50]. In addition, repeated heat shock operations might select for thermophilic *L. pneumophila* (able to grow above 50 °C [51]) or lead to thermo-acclimated *Legionella* post-heat shock [52].

Given that the heat shock treatment applied in this study represented the mild end of published guidelines, it would be of future interest to investigate a full range of heat shock procedures. While setting the water heater temperature at > 70 °C and flushing distal taps for 30 min would be expected to be more effective for OP control, this is not known for certain and it is plausible that selection of OPs could actually be stronger. In reality, the heat shock procedure a homeowner could reasonably achieve is to set the water heater temperature at the highest level—typically, 65.5 °C for residential electric water heaters [53] and 71.1 °C for residential gas water heaters [54]. Also, in large buildings with complex plumbing systems, it can be especially difficult to achieve and maintain target at-the-tap flushing temperatures. It would also be of future research interest to explore how the functional profiles of microbial communities are affected by heat shock protocols, as the present study largely focused on taxonomic responses.

## Conclusions

Here the effect of heat shock as a microbiota and *Legionella* control measure is evaluated. The mild heat shock procedure adopted in this study, i.e., maintaining at-the-tap flushing temperatures above 55 °C for approximately 30 min with water heater temperature set point at 60 °C, conferred little change in biofilm and bulk water microbiota at distal taps, where water is used and exposure occurs. Importantly and consistent with prior research, increased relative abundances of *L. pneumophila* and *V. vermiformis* were observed post-heat shock. Water heater temperature set point and water use frequency appeared to be promising long-term alternatives in terms of microbiota modification and *L. pneumophila* control.

## Additional files

Additional file 1: Table S1. Summary of heat shock treatment procedures. (XLS 30 kb)

Additional file 2: Figure S1. Principal Coordinate Analysis on distal tap microbiome composition. Figures are 3D Principal Coordinates Analysis based on weighted UniFrac distance matrices (rarefied to sequencing depth of 5, 200 for 100 times). Samples shown were distal tap ones. (TIFF 269 kb)

Additional file 3: Table S2. Distal tap water microbial dissimilarity patterns across sampling time. Data recorded in the form of “*R*^2^_Adonis_ (*P*_Adonis_); *P*_Betadisp_” (permutation = 999). (P ≥ 0.5). (XLS 30 kb)

Additional file 4: Figure S2. Chao 1 index of “heat shock” rig distal tap samples across time. Chao 1 index value is the average of 100 calculations based on rarefied OTU tables. (TIFF 200 kb)

Additional file 5: Figure S3. Gini index of “heat shock” rig distal tap samples across time. Gini index value is the average of 100 calculations based on rarefied OTU tables. (TIFF 199 kb)

Additional file 6: Figure S4. OTU counts pre- and post-heat shock comparison. All samples were from “heat shock” rig distal taps. Pre-heat shock included samples from 2-month pre- and immediately pre-heat shock, while post-heat shock included only samples from 2-month post-heat shock. Only OTUs detected at least once in either pre- or post-heat shock samples were included in this analysis. OTU counts (sequences per OTU) were first transformed as log10(OTU counts + 1). Linear regression was carried using transformed OTU counts. (TIFF 240 kb)

Additional file 7: Figure S5. Synergistic effect between pipe orientation and water use frequency. Figures are 3D Principal Coordinates Analysis based on weighted (top row) and unweighted (bottom row) UniFrac distance matrices (rarefied to sequencing depth of 5, 200 for 100 times). Samples shown were distal tap biofilm ones. (TIFF 272 kb)

Additional file 8: Figure S6. *Vermamoeba vermiformis* gene copy numbers in distal tap samples by qPCR. The gene copy numbers were log 10 transformed, i.e., gene copy number of X corresponds to log_10_(*X* + 1). (TIFF 225 kb)

Additional file 9: Figure S7. *Mycobacterium avium* gene copy numbers in distal tap samples by qPCR. Samples collected at 8-h post-heat shock (+ 8 h) was not included. The gene copy numbers were log 10 transformed, i.e., gene copy number of X corresponds to log_10_(*X* + 1). (TIFF 211 kb)

Additional file 10: Table S3. Simplified model for estimating proportion of directly impacted water along time. “Remained” is the remaining “original” heat shocked water within water heater and recirculating line. Assumptions include: water heater and recirculating line is a complete mixed system with no (little) change between distal pipe and recirculating line; no microbial death or growth; no interference with biofilm; only considering physical mixing. Recirculating line sampling not accounted for. (XLS 58 kb)

Additional file 11: Figure S8. *Legionella pneumophila* gene copy numbers in distal tap samples by qPCR. The gene copy numbers were log 10 transformed, i.e., gene copy number of X corresponds to log_10_(*X* + 1). (TIFF 223 kb)
