# Supplementary figures and images for: Effect of heat shock on hot water plumbing microbiota and Legionella pneumophila control
Source: Microbiome. 2018 Feb 9;6:30. doi: 10.1186/s40168-018-0406-7 (PMC5807837; doi:10.1186/s40168-018-0406-7)

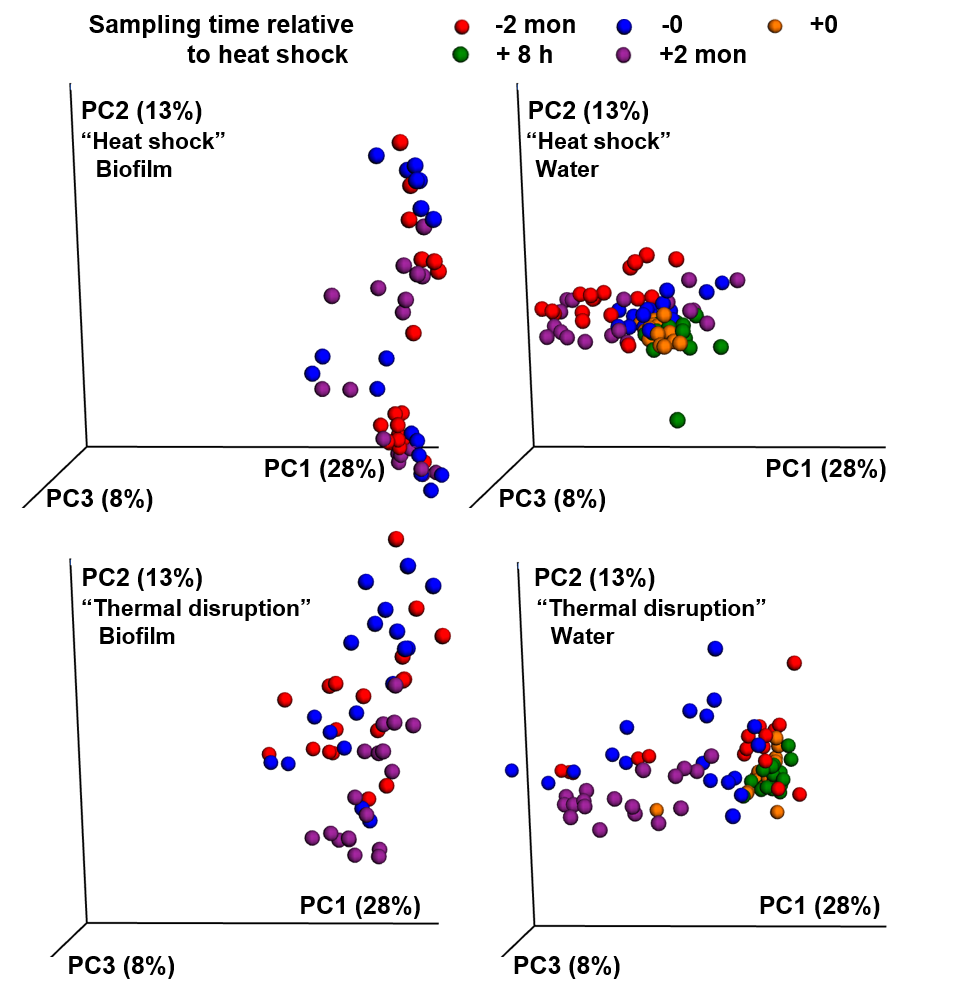

Supplement: Supplementary file 2 — Figure S1. Principal Coordinate Analysis on distal tap microbiome composition. Figures are 3D Principal Coordinates Analysis based on weighted UniFrac distance matrices (rarefied to sequencing depth of 5, 200 for 100 times). Samples shown were distal tap ones. (TIFF 269 kb) [file 40168_2018_406_MOESM2_ESM.tif]

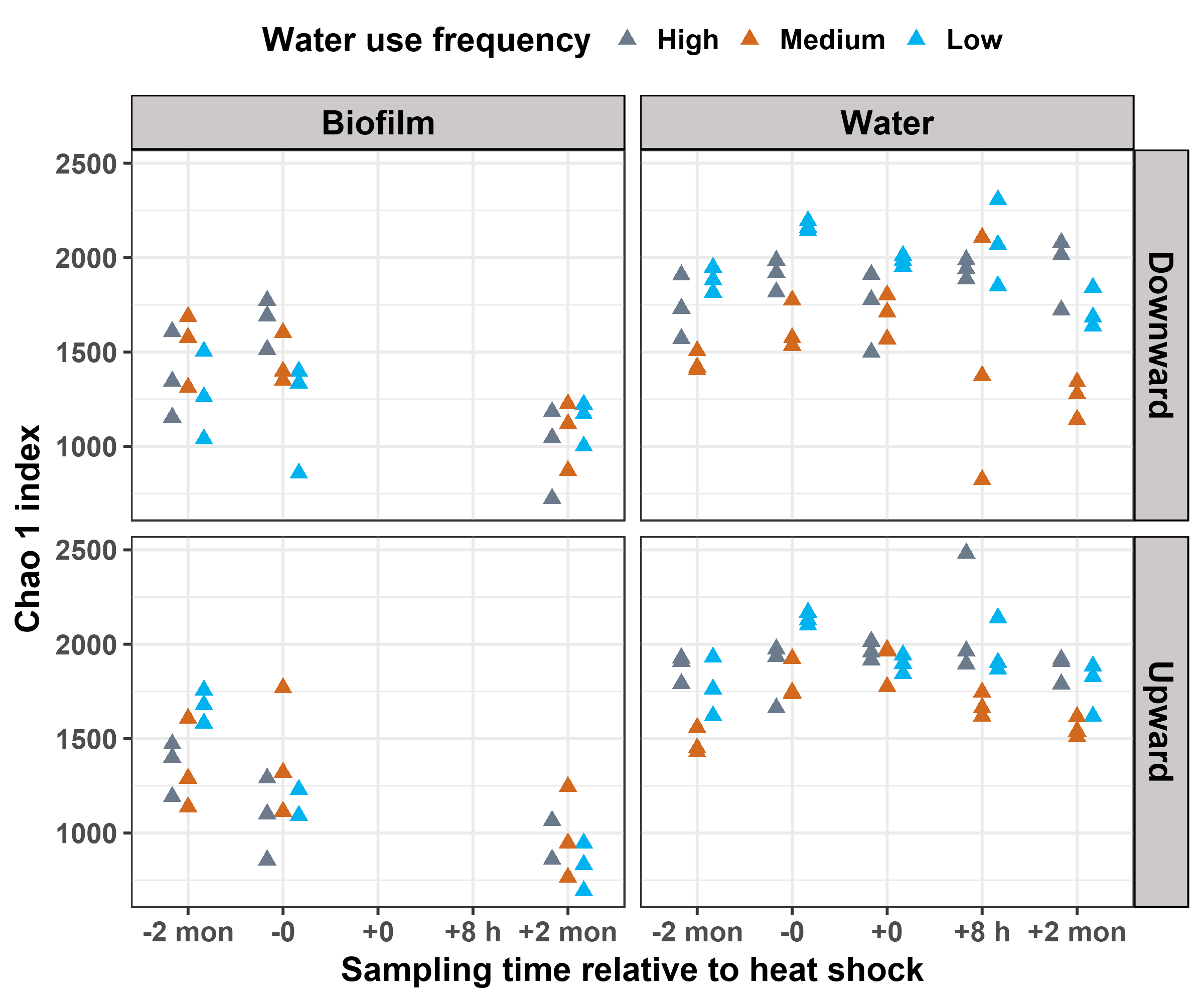

Supplement: Supplementary file 4 — Figure S2. Chao 1 index of “heat shock” rig distal tap samples across time. Chao 1 index value is the average of 100 calculations based on rarefied OTU tables. (TIFF 200 kb) [file 40168_2018_406_MOESM4_ESM.tiff]

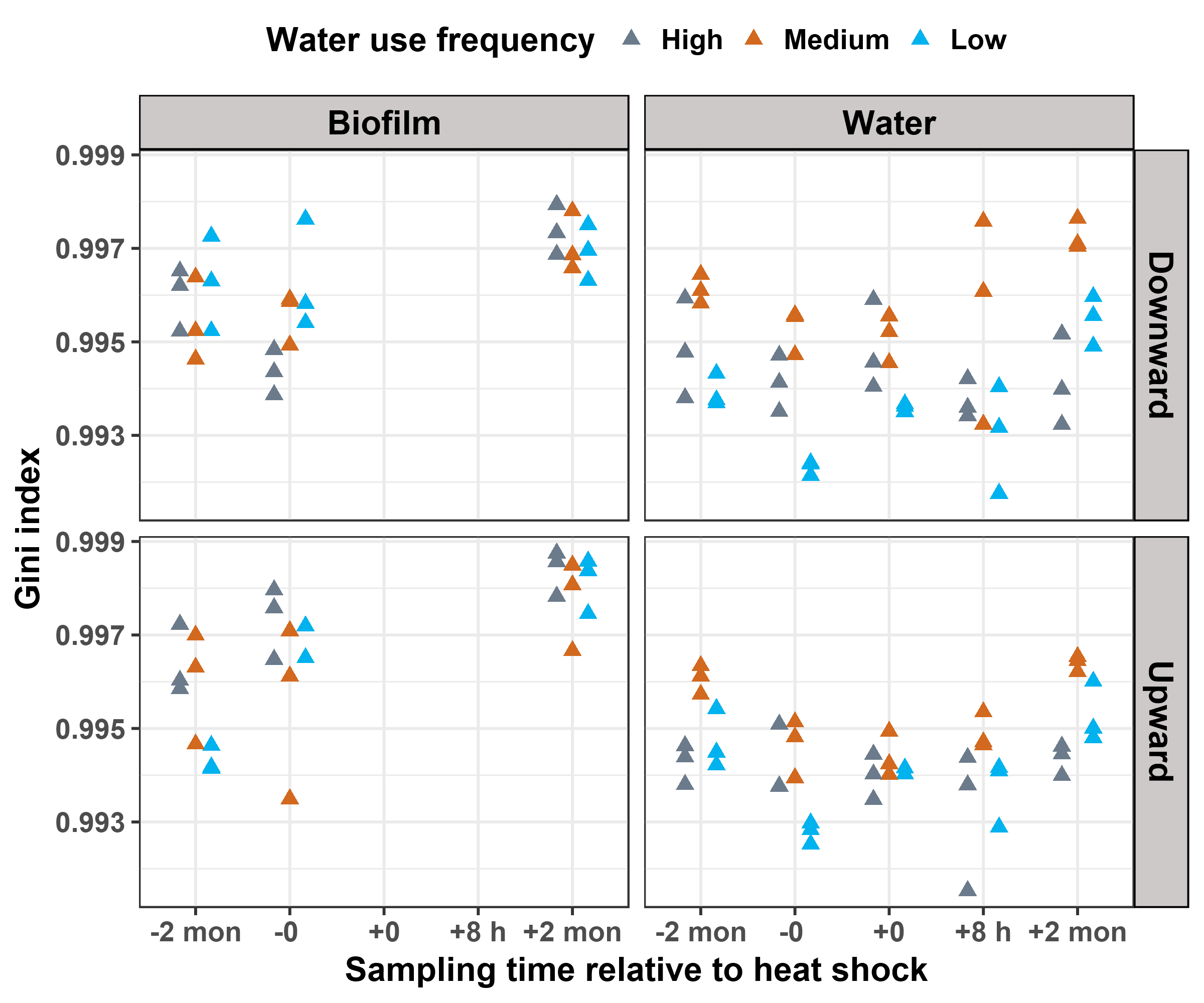

Supplement: Supplementary file 5 — Figure S3. Gini index of “heat shock” rig distal tap samples across time. Gini index value is the average of 100 calculations based on rarefied OTU tables. (TIFF 199 kb) [file 40168_2018_406_MOESM5_ESM.tiff]

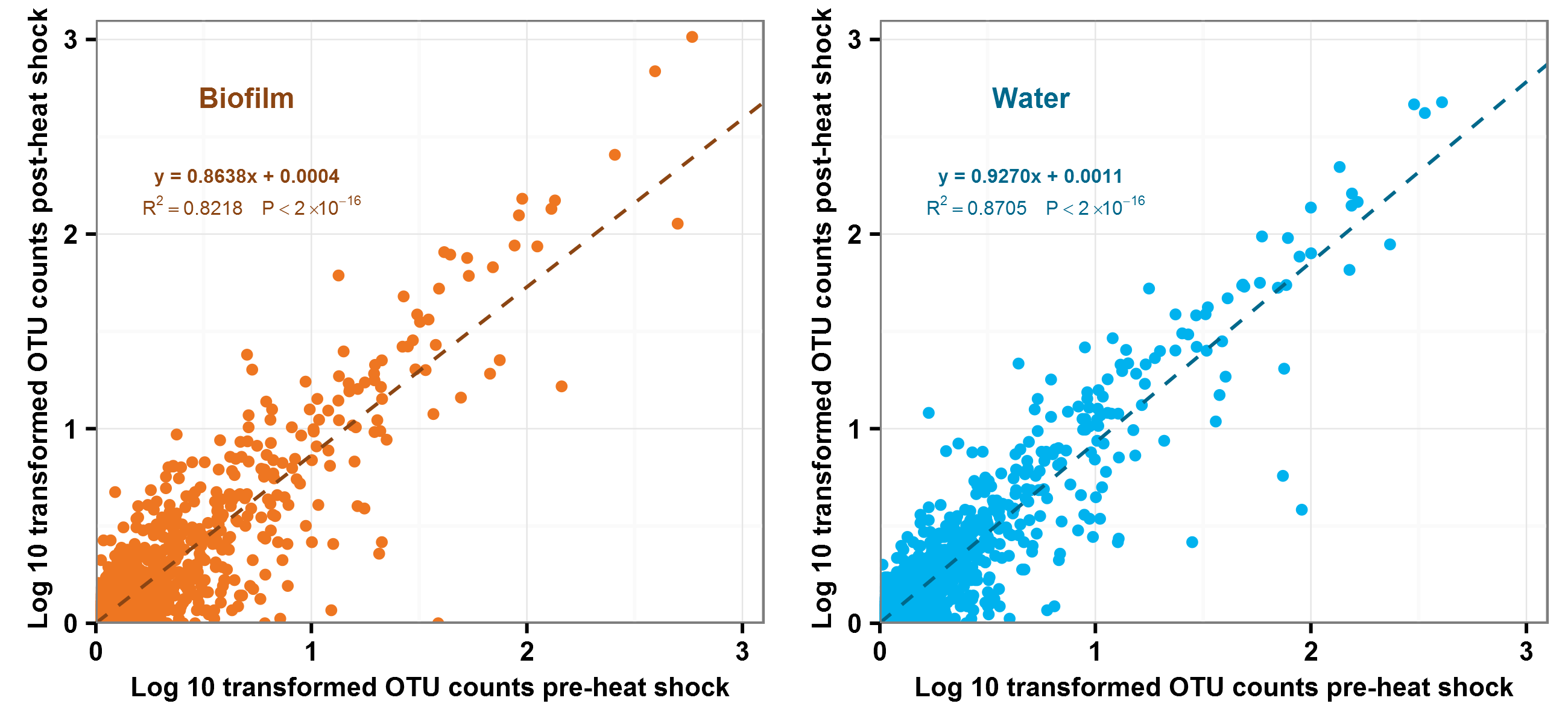

Supplement: Supplementary file 6 — Figure S4. OTU counts pre- and post-heat shock comparison. All samples were from “heat shock” rig distal taps. Pre-heat shock included samples from 2-month pre- and immediately pre-heat shock, while post-heat shock included only samples from 2-month post-heat shock. Only OTUs detected at least once in either pre- or post-heat shock samples were included in this analysis. OTU counts (sequences per OTU) were first transformed as log10(OTU counts + 1). Linear regression was carried using transformed OTU counts. (TIFF 240 kb) [file 40168_2018_406_MOESM6_ESM.tiff]

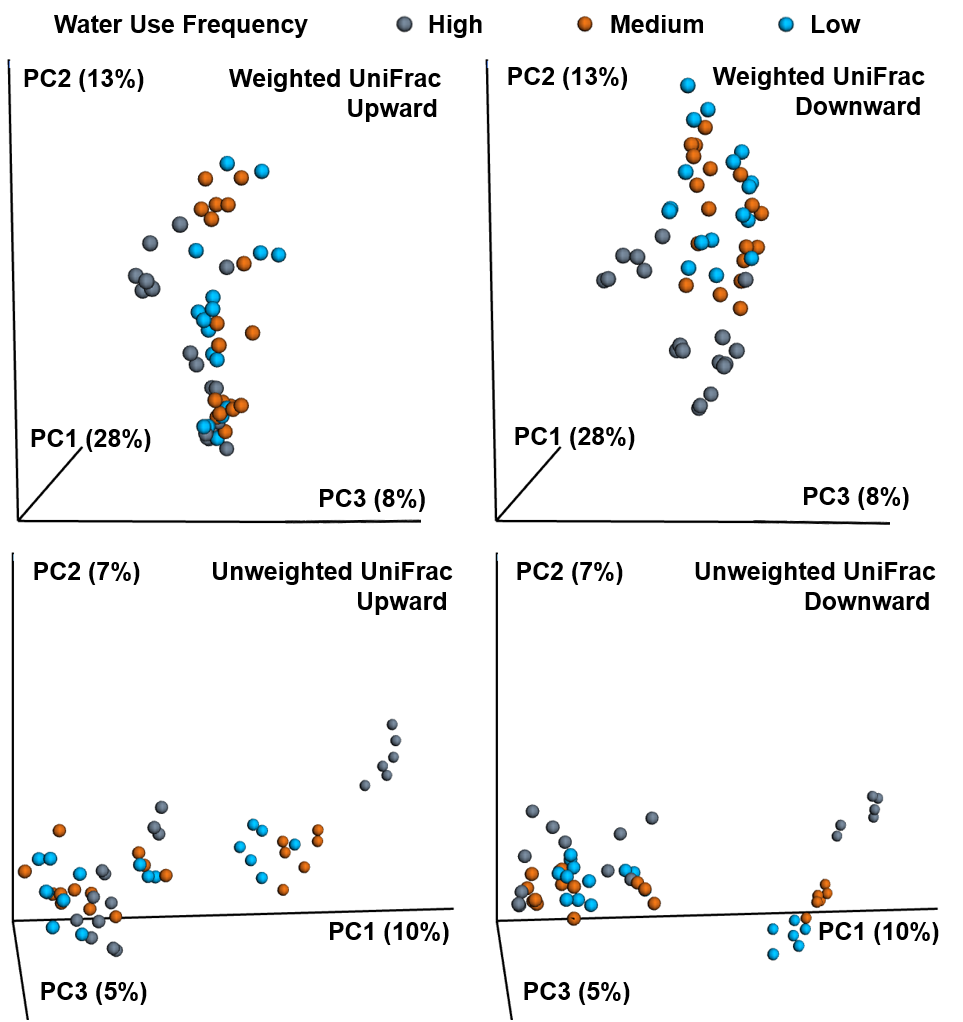

Supplement: Supplementary file 7 — Figure S5. Synergistic effect between pipe orientation and water use frequency. Figures are 3D Principal Coordinates Analysis based on weighted (top row) and unweighted (bottom row) UniFrac distance matrices (rarefied to sequencing depth of 5, 200 for 100 times). Samples shown were distal tap biofilm ones. (TIFF 272 kb) [file 40168_2018_406_MOESM7_ESM.tif]

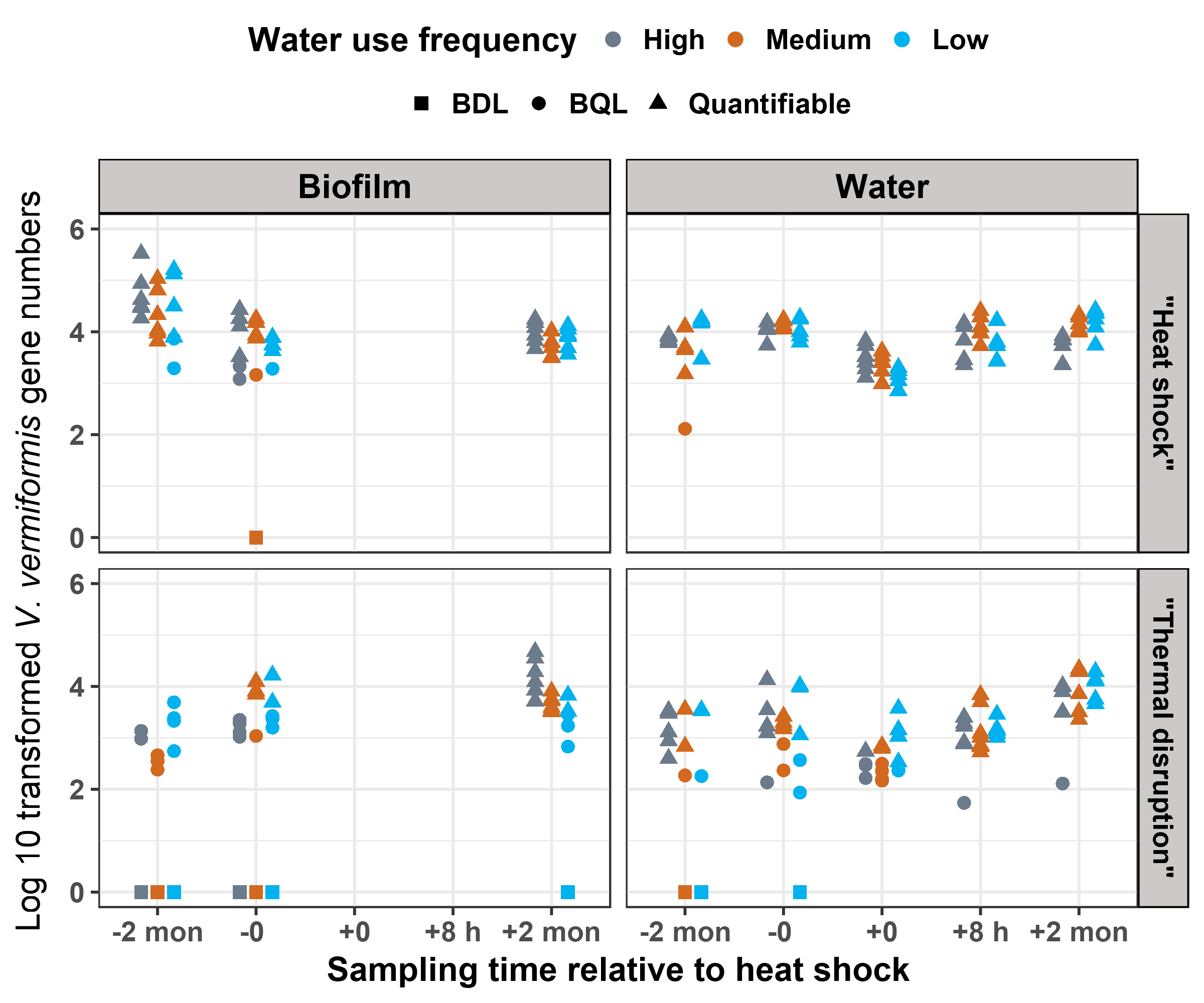

Supplement: Supplementary file 8 — Figure S6. Vermamoeba vermiformis gene copy numbers in distal tap samples by qPCR. The gene copy numbers were log 10 transformed, i.e., gene copy number of X corresponds to log10(X + 1). (TIFF 225 kb) [file 40168_2018_406_MOESM8_ESM.tiff]

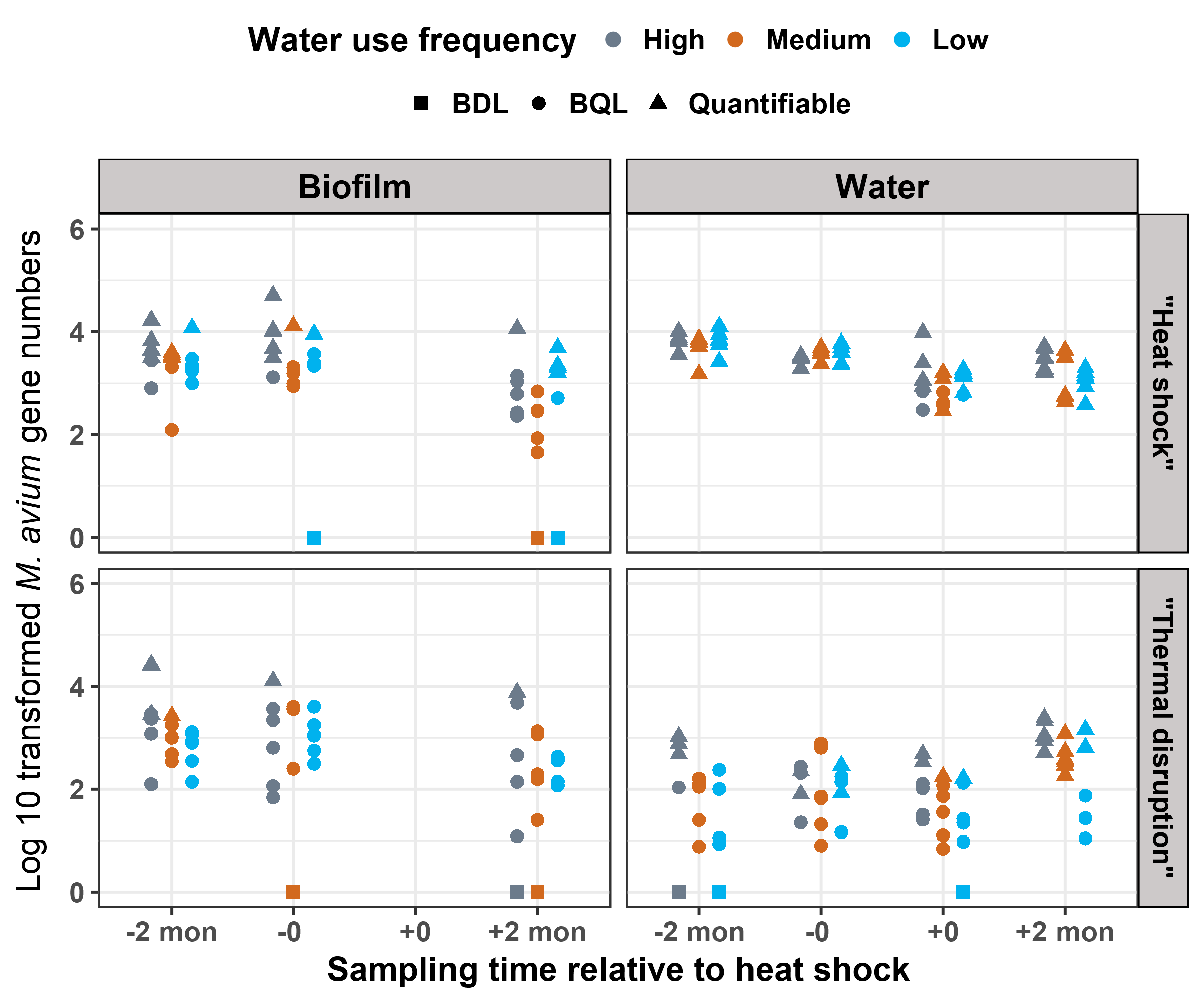

Supplement: Supplementary file 9 — Figure S7. Mycobacterium avium gene copy numbers in distal tap samples by qPCR. Samples collected at 8-h post-heat shock (+ 8 h) was not included. The gene copy numbers were log 10 transformed, i.e., gene copy number of X corresponds to log10(X + 1). (TIFF 211 kb) [file 40168_2018_406_MOESM9_ESM.tiff]

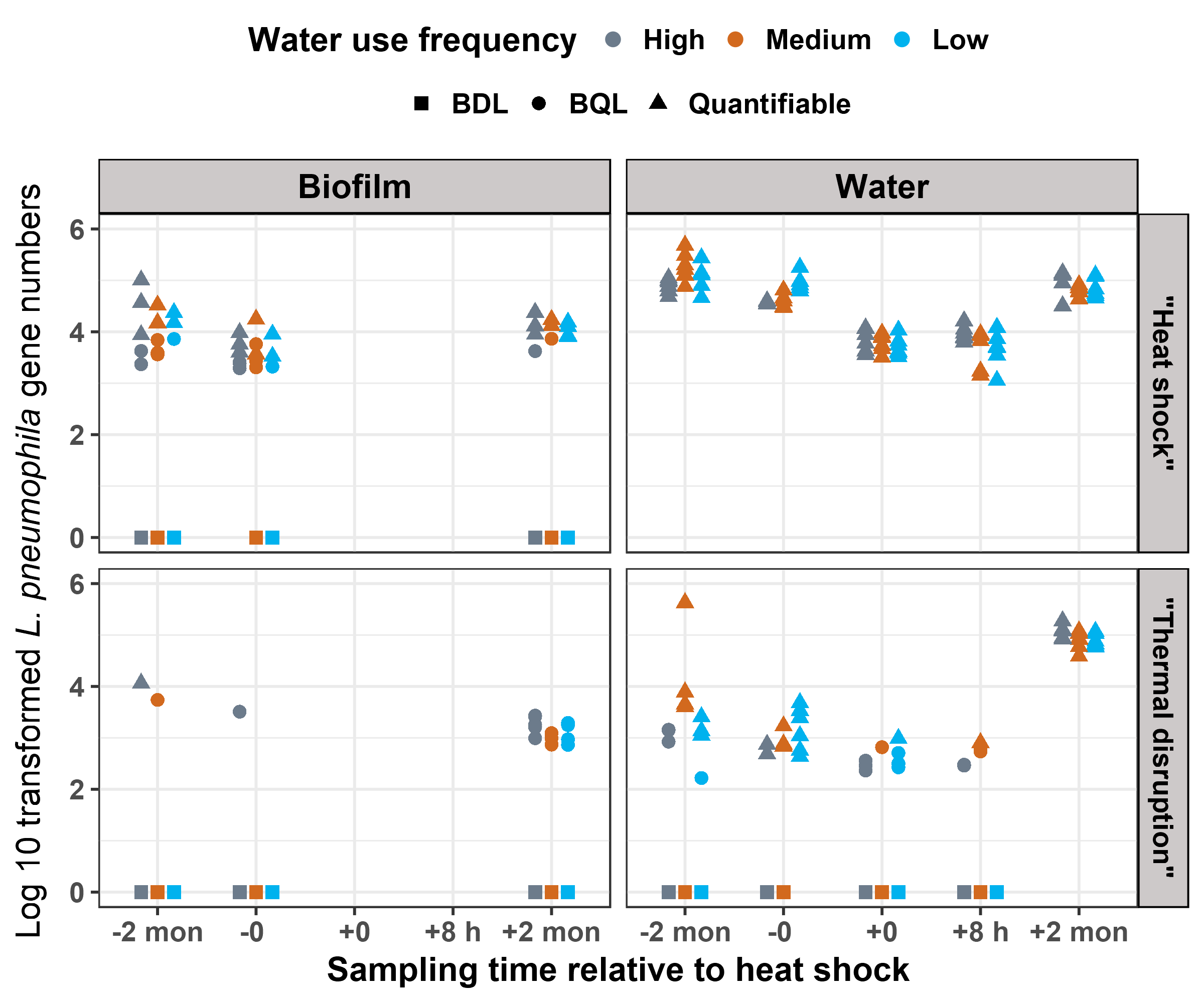

Supplement: Supplementary file 11 — Legionella pneumophila gene copy numbers in distal tap samples by qPCR. The gene copy numbers were log 10 transformed, i.e., gene copy number of X corresponds to log10(X + 1). (TIFF 223 kb) [file 40168_2018_406_MOESM11_ESM.tiff]
